# The Synergistic Impact of Excessive Alcohol Drinking and Cigarette Smoking upon Prospective Memory

**DOI:** 10.3389/fpsyt.2016.00075

**Published:** 2016-04-27

**Authors:** Anna-Marie Marshall, Thomas Heffernan, Colin Hamilton

**Affiliations:** ^1^Department of Psychology, Northumbria University, Newcastle upon Tyne, UK

**Keywords:** excessive drinking, smoking, synergistic, prospective memory, CAMPROMPT

## Abstract

The independent use of excessive amounts of alcohol or persistent cigarette smoking have been found to have a deleterious impact upon Prospective Memory (PM: remembering future intentions and activities), although to date, the effect of their concurrent use upon PM is yet to be explored. The present study investigated the impact of the concurrent use of drinking excessive amounts of alcohol and smoking cigarettes (a “Polydrug” group) in comparison to the combined effect of the single use of these substances upon PM. The study adopted a single factorial independent groups design. The Cambridge Prospective Memory Test (CAMPROMPT) is a test of both time-based and event-based PM and was used here to measure PM. The CAMPROMPT was administered to 125 adults; an excessive alcohol user group (*n* = 40), a group of smokers who drink very little alcohol (*n* = 20), a combined user group (the “Polydrug” group) who drink excessively and smoke cigarettes (*n* = 40) and a non-drinker/low alcohol consumption control group (*n* = 25). The main findings revealed that the Polydrug users recalled significantly fewer time-based PM tasks than both excessive alcohol users *p* < 0.001 and smokers *p* = 0.013. Polydrug users (mean = 11.47) also remembered significantly fewer event-based PM tasks than excessive alcohol users *p* < 0.001 and smokers *p* = 0.013. With regards to the main aim of the study, the polydrug users exhibited significantly greater impaired time-based PM than the combined effect of single excessive alcohol users and cigarette smokers *p* = 0.033. However, no difference was observed between polydrug users and the combined effect of single excessive alcohol users and cigarette smokers in event-based PM *p* = 0.757. These results provide evidence that concurrent (polydrug) use of these two substances has a synergistic effect in terms of deficits upon time-based PM. The observation that combined excessive drinking and cigarette smoking leads to a greater impairment in time-based PM may be of paramount importance, given the key role PM plays in everyday independent living.

## Introduction

Tobacco and alcohol are two of the most widely used drugs in the Western world and are responsible for a large proportion of harm (1). These two drugs are often used concurrently (2, 3); yet, there remains a paucity of research in relation to their combined effects. The relationship between these drugs is complex and not presently well understood; this is surprising given the synergistic health risks posed by such polydrug use (4). Previous research suggests that chronic use of large amounts of alcohol and cigarette smoking are independently associated with a variety of cognitive impairments. For example, studies that have examined the effects of alcohol on cognitive performance and have shown that drinking excessively impairs Working Memory (WM), which is responsible for the manipulation and maintenance of information across a short period of time; for example, remembering someone’s phone number while driving a car and concentrating on the road ahead (5, 6) as well as Executive Function (EF), which is an umbrella term used to describe a set of resources that are responsible for the management of cognitive functions, including WM and attention; for example, being able to pay attention to a task despite having distractions all around you (7, 8). More recently, excessive alcohol use has been associated with poorer performance in Prospective Memory (PM), which refers to the cognitive ability to carry out planned intentions/actions at a future point in time (9–11). Excessive alcohol drinking is defined as either drinking in excess of the current cut-off limits for safe drinking, which are 14/21 U of alcohol per week for females and males, respectively (12, 13). It should be noted that a UK unit (8 g ethanol) contains 0.343 US fluid ounces of ethanol. Chronic cigarette smoking has also been associated with deficits in these domains, including WM (14, 15), EF (16, 17), and PM (18–20). PM is seen as an important part of everyday remembering, since it is responsible for planning and remembering future activities, such as remembering to meet with friends at a pre-specified time and location, remembering to take an important medication on time, or remembering to turn up for a meeting; in this respect, it is seen as essential for independent living (21).

Prospective memory involves both time-based (an action that is carried out after a specific time period has elapsed; for example, remembering to take an important medication after a specific time has elapsed) and event-based (an action executed as the result of an environmental cue; for example, remembering to pass on a message to someone whom you meet in the street). It has been suggested that the subtypes rely upon different cognitive mechanisms; event-based involves spontaneous retrieval while time-based requires attentional monitoring and as such time-based may be more reliant upon executive resources (22). There is evidence to suggest that event-based and time-based PM are, at least in part, separable. For example, time-based, but not event-based PM deficits were found in a patient with bilateral frontal lobe infarcts (23); whereas Parkinson’s disease patients have been found to be impaired on the event-based, but not on time-based, PM tasks (24), suggesting a dissociation between the two processes. In addition, research using Positron Emission Tomography (PET) brain imaging has found evidence of differential involvement of prefrontal regions in time-based and event-based PM (25). Although there is a paucity of research focusing upon the combined effect of alcohol and tobacco use upon cognition and memory, there is some. Evidence in relation to the interactive effect between excessive alcohol use and smoking is evident in other areas of cognition. Cigarette smoking has been found to exacerbate cognitive deficits in those who drink alcohol excessively, including memory deficits, one’s ability to think quickly and efficiently, as well as on problem solving tasks (26). Recently, evidence has shown that alcohol-dependant individuals who smoke cigarettes show greater neuropsychological damage than those who do not smoke (27); observing decreased cortical thickness in the polydrug users, with greater thinning in frontal areas of the cortex (a key brain region involved in PM). The combined effect of smoking and alcohol has also recently been linked with faster cognitive decline in such polydrug users, compared with alcohol users alone (28), indicating that cigarette smoking and excessive alcohol use may act in synergy to cause increased cognitive decline. In addition to this greater deficits in EF [believed to rely on the same cognitive processes as PM: (29)] have been found in such polydrug user groups compared to alcohol users alone. Given the cumulative evidence that the combined use of excessive amounts of alcohol and cigarette smoking may damage pre-frontal regions of the brain and may accelerate declines in cognitive processes such as EF, it is possible that the combined use of these two substances may exacerbate declines in PM when compared with the single use of these substances.

The main aim of the study is to explore whether the combined (polydrug) effect of consuming excessive amounts of alcohol and smoking cigarettes is greater than the sum of their independent effects. This will be achieved by comparing the added effects of both single user groups (an excessive drinking group and a cigarette smoking group) with a polydrug group (those who drink alcohol excessively and smoke cigarettes) in order to determine whether there is a significant difference between these with regards PM. This should provide insight as to whether the combination of these two substances has an additive or synergistic impact upon PM function. Since PM involves both time and event-based tasks, the Cambridge Prospective Memory Test (CAMPROMPT) was utilized here as a measure of both time and event-based PM.

## Materials and Methods

### Design

An existing groups design was employed comparing four groups: (1) an “Excessive Alcohol” group who drank excessive amounts of alcohol but who did not smoke cigarettes; (2) a “Cigarette Smokers” group who smoked cigarettes on a regular/daily basis and drank very little alcohol; (3) a “Polydrug” group who drank excessively and smoked cigarettes on a regular/daily basis; and (4) a “Control” group who drank low amounts of alcohol who had never smoked cigarettes. Excessive alcohol drinking was classified as those individuals who drank in excess of the current cut-off limits for safe drinking, which are 14/21 U of alcohol (females and males respectively) per week, as described in the Section “Introduction.” The dependent measures included both time-based and event-based CAMPROMPT scores.

### Participants

One-hundred and twenty-five unpaid volunteers were recruited as participants through opportunity sampling, which involved taking a sample of people who responded *via* advertisements about the study and who fit the criteria for which the researchers were looking. The inclusion criteria was anyone who fell in to one of the four groupings identified above; therefore any person who was either an excessive drinker (drinking above the 14/21 U of alcohol per week described above), a regular cigarette smoker who drank very little (ranging from 0 to 7 U of alcohol per week), a polydrug user who drank excessively and smoked cigarettes regularly, or a non-smoker who consumed very little (if any) alcohol. The study was advertised widely around the university, and the inclusion criteria were made clear so that we could ensure recruitment to all of the groups. Anyone who reported using other substances, such as cannabis, ecstasy, heroin, cocaine, “legal highs,” etc., were excluded from the study. Anyone who reported having previously suffered from/were currently suffering from, a clinical disorder (such as amnesia, depression, or substance dependence), were excluded from the study. The age range of participants was between 18 and 43 years. Participants were allocated to a group based upon their alcohol and cigarette use. Excessive alcohol was determined by the participant’s weekly alcohol usage (regardless of any specific pattern of drinking, such as “binge drinking”), which was whether they exceeded the 14/21 U of alcohol per week for females and males, respectively (12, 13). The Excessive Alcohol group contained 40 participants (25 females) who had never smoked, and their mean alcohol intake per week was 25.9 U (SD 8.60). The Cigarette Smokers group consisted of 20 participants (14 females) who smoked on a regular/daily basis, but did not consume alcohol on a regular basis and drank low amounts of alcohol; they smoked on average 69.3 cigarettes per week (SD 47.7). The Polydrug group contained 40 participants (16 females) who smoked cigarettes on a regular/daily basis and drank excessively; their mean alcohol consumption was 26.5 U per week (SD 6.88), and their mean cigarettes usage per week was 52.5 cigarettes (SD 27.2). The Control group consisted of 25 participants (19 females) who were low-dose alcohol users/non-users who did not smoke; their mean alcohol consumption per week was 1.46 U (SD 2.38). The Excessive Alcohol and Polydrug groups did not differ in terms of the amount of alcohol they consumed per week, nor in terms of the years spent drinking alcohol or their last alcohol use in hours. The Cigarette Smokers and Polydrug groups did not differ in terms of the amount of cigarettes they smoked per week or in terms of their last cigarette use in hours, but the Cigarette Smokers group had been smoking for longer than the Polydrug group. The distribution of male and female participants between the groups did differ significantly. See the Section “Results” for analysis of these non-memory measures.

### Measures

The Cambridge Prospective Memory Test (CAMPROMPT) is a valid and reliable measure of time-based and event-based PM (30) and was utilized in the current study as an objective measure of both time and event-based PM. The test consists of three time-based tasks, which require the participant to carry out a task at a specific time; a clock was available for them to monitor the time (for example, “In seven minutes, I would like you to change the pen you are using”) and three event-based tasks, which require the participant to carry out a task in response to a cue (for example, “When you come to a quiz question about ‘Eastenders’ I would like you to give me this book”). The time-based and event-based tasks were to be remembered while completing a set of distracter tasks comprising a set of puzzles. Points were scored for each of the six tasks and the scoring per task ranged from 6 (where the participant completed task unaided) to 0 (where they have failed to complete task), with points between these two on the scale for tasks completed with some prompting from the researcher. Two types of PM scores were obtained: a time-based PM score (out of a maximum of 18) and an event-based PM score (out of a maximum of 18), with the higher score reflecting a more proficient PM.

Alcohol use, smoking, and other drug use were measured using a modified version of the University of East London Recreational Drug Use Questionnaire [RDUQ: (31)]. This questionnaire asked the participant to report their drinking and smoking pattern over a typical week, including quantities and the number of units of alcohol/cigarettes, hours since last use and years spent using alcohol/cigarettes. Participants were further asked to state any other drug use, such as cannabis and ecstasy, and amount and frequency of use.

### Procedure

Prior to commencement, the research protocol was approved by the School of Health and Life Sciences Ethics Committee at Northumbria University. All testing was carried out in a laboratory setting, taking approximately 30 min to complete. Participation was voluntary. The CAMPROMPT was administered first, in which participants were asked to complete a set of puzzles and quizzes, while being asked by the researcher to remember to carry out the time-based and event-based memory tasks; this lasted approximately 25 min. Participants were then asked to complete the RDUQ questionnaire, which took only a few minutes. Upon completion, participants were debriefed, any questions they had were answered and they were given the opportunity to withdraw their data from the study (none did so).

## Results

In order to identify that the Polydrug user group was appropriately matched to the respective single drug user groups, a series of one-way ANOVAs were applied to the data comparing appropriate groups on alcohol use and smoking. The Excessive Alcohol group and Polydrug group were compared on the amount of alcohol consumed per week, the number of years spent drinking alcohol, and the number of hours since they last drank alcohol (see Table 1 for the means and standard deviations (SDs) for these measures across the groups). The Cigarette Smokers group and Polydrug group were compared on the number of cigarettes smoked per week, the number of years spent smoking, and the number of hours since their last cigarette was used (see Table 1 for the means and SDs for these measures across the groups). The analyses revealed no significant differences between the Excessive Alcohol and Polydrug groups in terms of the number of alcohol units consumed per week [*F*(1,78) = 0.150, *p* = 0.699], the number of years spent drinking alcohol [*F*(1,78) = 0.153, *p* = 0.697], and hours since they last drank alcohol [*F*(1,78) = 0.414, *p* = 0.522]. No significant difference was observed between the Cigarette Smokers group and Polydrug Group in terms of the amount of cigarettes smoked per week [*F*(1,58) = 3.001, *p* = 0.089] and number of hours since their last cigarette [*F*(1,58) = 0.034, *p* = 0.855]; however there was a significant difference in terms of the number of years for which participants had smoked cigarettes [*F*(1,58) = 8.624, *p* = 0.005] – with the Cigarette Smokers group having smoked for longer than the Polydrug group. In summary, there were no significant between group differences between excessive alcohol use and smoking pattern for the potential confounding variables, other than years spent smoking.

**Table 1:** **Means (and SDs) for all measures across each drug user group**.

	Excessive alcohol (*n* = 40)	Cigarette smokers (*n* = 20)	Polydrug (*n* = 40)
Age	22.30 (4.10)	27.15 (6.80)	22.55 (4.16)
Alcohol units per week	25.90 (8.60)	0.55 (1.42)	26.58 (6.88)
Years drinking alcohol	6.48 (4.39)	1.30 (3.70)	6.84 (3.88)
Hours since last alcohol	90.95 (73.30)	45.60 (107.02)	105.68 (124.83)
Cigarettes per week	0.00 (0.00)	69.30 (47.79)	52.55 (27.23)
Years smoking cigarettes	0.00 (0.00)	10.95 (9.15)	5.99 (3.98)
Hours since last cigarette	0.00 (0.00)	7.70 (12.27)	8.49 (17.01)
CAMPROMPT event	14.27 (2.35)	13.85 (2.52)	11.47 (3.34)
CAMPROMPT time	14.67 (2.41)	12.30 (2.39)	9.77 (3.99)

A multivariate MANOVA was applied to the data in order to identify whether CAMPROMPT event-based and time-based differences existed between the Polydrug group and the two single user groups. This revealed a significant effect of group on the dependent measures [Wilk’s Lambda = 0.636, *F* (4,192) = 12.182, *p* < 0.001; ηp^2^ = 0.20]. This analysis indicated a significant effect associated with the time-based CAMPROMPT [*F*(2,97) = 24.367, *p* < 0.001, ηp^2^ = 0.33] and a significant effect associated with the event-based measure [*F*(2,97) = 10.799, *p* < 0.001, ηp^2^ = 0.18]. Bonferroni adjusted *post hoc* analysis revealed that with regard to event-based PM, the Polydrug group (mean = 11.47; SD 3.34) remembered significantly fewer actions than Excessive Alcohol group (mean = 14.27; SD 2.35), *p* < 0.001, and that the Polydrug users also remembered significantly fewer event-based actions than the Cigarette Smokers group (mean = 13.85; SD 2.52), *p* = 0.008. With regard to time-based PM, the Polydrug group (mean = 9.77; SD 3.99) remembered significantly fewer PM actions than Excessive Alcohol group (mean = 14.67; SD 2.41), *p* < 0.001, and that the Polydrug users also remembered significantly fewer time-based items than the Cigarette Smokers group (mean = 12.30; SD 2.39), *p* = 0.012.

**Figure 1:**
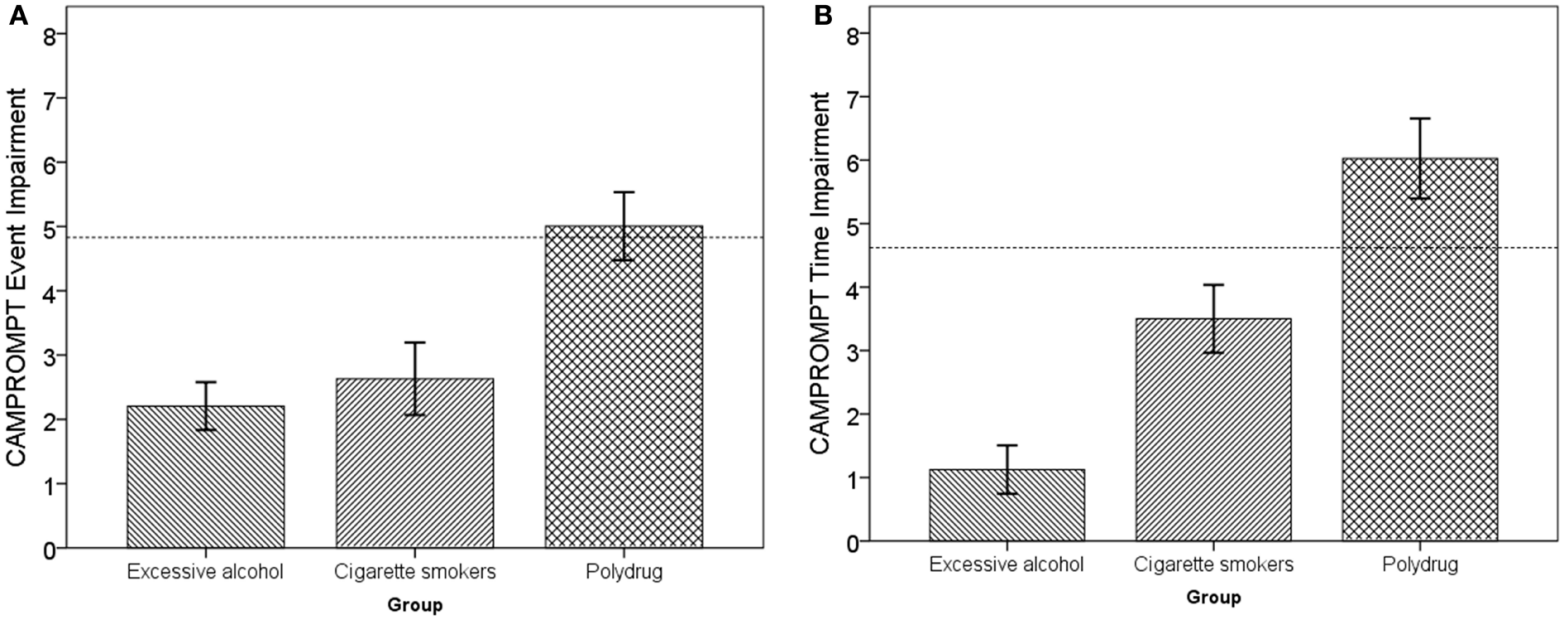
**(A)** CAMPROMPT event-based impairment as a function of user group. **(B)** CAMPROMPT time-based impairment as a function of user group.

To address the main aim of whether the combined (polydrug) effect of consuming excessive amounts of alcohol and smoking cigarettes is greater than the sum of their independent effects, the CAMPROMPT impairment of the Polydrug Group was contrasted with the combined impairment of the two single user groups (the Excessive Alcohol group and Cigarette Smokers group). Specific impairment of the two single user groups was identified by comparing their mean performance with that of the Control group performance on both CAMPROMPT measures. In the event-based CAMPROMPT measure, the Control mean performance was 16.48 (SD 2.48), and the Excessive Alcohol and the Cigarette Smokers group achieved 14.27 and 13.85, respectively. Thus, the specific impairments for these two user groups were; Excessive Alcohol, 16.48 − 14.27 = 2.21, and Cigarette Smokers, 16.48 − 13.85 = 2.63. The combined event-based impairment level was therefore = 2.21 + 2.63 = 4.84. This combined baseline impairment value is shown in Figure 1A below by the horizontal dashed line.

In the time-based CAMPROMPT measure, the Control mean performance was 15.80 (SD 2.00). Thus, the specific impairments for these two user groups were; Excessive Alcohol, 15.80 − 14.67 = 1.13, and Cigarette Smokers, 15.80 − 12.30 = 3.50. The combined time-based impairment level was therefore 4.63, and this combined impairment value is shown by the horizontal dashed line in Figure 1B below. In order to identify whether the Polydrug impairment (in comparison to the control group performance) was greater than the combined single user deficits, two one-sample *t*-tests were conducted with the respective combined impairments as the criteria. In relation to event-based PM, there was no significant difference between the Polydrug groups’ impairment and the combined single user impairment, [*t*(39) = −0.312, *p* = 0.757]. In relation to time-based PM, the Polydrug groups’ impairment was significantly greater than the combination of single user impairments [*t*(39) = −2.243, *p* = 0.031].

In summary, significantly more time-based and event-based PM errors were made by the Polydrug user group in comparison to both Excessive Alcohol user group and Cigarette Smoker groups. Importantly, further analysis revealed that the polydrug group also made significantly more PM errors that the combined effect of excessive alcohol and cigarette smoking in time-based PM, although not in event-based PM.

## Discussion

The present study explored whether the combined (polydrug) effect of consuming excessive amounts of alcohol and smoking cigarettes is greater than the sum of their independent effects. This was achieved by comparing the added deficits for each of the single user groups (the Excessive alcohol drinkers and cigarette smokers group) with the polydrug group (those who both drink alcohol excessively and smoke cigarettes) to determine whether there was a significant difference between these two in terms of time-based and event-based PM function using the Cambridge Prospective Memory Test (CAMPROMPT) as the main measure of PM. With regards to this aim, the prospective memory (PM) deficits observed in the Polydrug group (those who drank excessive amounts of alcohol and smoked cigarettes) was found to be greater than the combined deficits of the two single user groups (the Excessive alcohol group and the Cigarette Smoker group) in relation to time-based PM, suggesting a synergistic interactive effect rather than an additive interactive effect of these two substances. No such effect was observed in relation to event-based PM. These effects were found after observing no significant differences between these groups in the amount of alcohol consumed per week, the number of years drinking alcohol, the number of hours since they last drank alcohol, the number of cigarettes smoked per week, and in terms of the number of hours since last cigarette. These findings indicate that, using both substances together produces greater deficits than single use of either substance and furthermore, the interaction between excessive amounts of alcohol and cigarettes produces greater deficits upon time-based PM than the sum of their separate effects – suggesting a synergistic effect of combined excessive drinking and smoking upon time-based PM. These findings firstly lend support to the body of research, which has previously found that drinking alcohol and smoking cigarettes separately, is associated with impaired PM (9–11, 18–20), but extends this by observing a synergistic effect of the combined use of excessive amounts of alcohol and cigarette smoking on time-based PM deficits when compared with their single separate use. Although smoking has been found to exacerbate cognitive deficits in excessive alcohol users in the past (26–28), the current study is the first to show this effect for prospective remembering. Given the importance of PM to everyday activities (21), this finding may be important in terms of its suggestion that everyday memory (of which PM is a very good example) may be compromised by the combination of excessive drinking and cigarette smoking in a non-clinical population.

Although this study has demonstrated synergistic effects of excessive drinking and smoking upon PM, the putative underlying damage to the mechanisms underpinning such deficits remains unclear. PM is believed to be a function underpinned by multiple cognitive processes rather than being a single construct in its own right; thus, it is difficult to identify a specific region or mechanism in the brain that may account for the PM deficits caused by excessive alcohol use, smoking, and polydrug use. However, both excessive alcohol use and smoking have been found to impair frontal lobe tasks such as EF (16, 32). Given previous clinical evidence that excessive alcohol users who also smoke show decreased cortical thickness, with greater thinning in frontal areas of the cortex (a key brain region involved in PM) compared with heavy drinking who do not smoke (27), it is possible that the deficits in PM found in the polydrug group in the current study may be the result of frontal lobe dysfunction. Given that the combination of drinking heavily and smoking cigarettes also leads to significant deficits in EF (28), which is heavily involved in frontal lobe resources and is believed to underpin PM (29), this lends further support to the notion that it is the frontal lobe region that is affected by the combined use excessive alcohol drinking and cigarette smoking. This could be explored further by the use of brain imaging (such as PET) alongside a measure of PM (such as CAMPROMPT) in order to observe the degree of frontal lobe activity during the PM task in the polydrug users compared with suitable controls. Again, one must be cautious given the evidence from neuroimaging studies which have also implicated other regions, such as the hippocampus and thalamus in PM (33–35). It is therefore possible that any putative damage as a result of polydrug use may well be indicative of damage that is not confined to the frontal region itself; again, brain imaging techniques could be used in combination with the CAMPROMPT in order to elucidate the links between polydrug use, PM deficits and any underlying neuropsychological damage. The fact that this synergistic effect (i.e., the finding that polydrug user group showed greater PM deficits than the combined deficits of both single user groups) was evident only for time-based PM task is explicable in terms of time-based PM being more reliant upon frontal lobe processes (21), and therefore, if the frontal lobes are being damaged/depleted by the combination of drinking excessively and smoking cigarettes, then one would expect to find this for the time-based tasks and not for the event-based tasks (as was the case in the findings of the current study). This suggests that event-based PM may operate on a different neural network than that of time-based PM, a point that could be pursued in future research. It may also be worth noting that, since nicotine is seen to act as neuroprotective (36), the contributing factor of cigarette smoking to this synergistic effect on PM must come from the toxins contained in cigarette smoke and inhaled by smokers, these toxins combined with excessive alcohol use must act together to damage or deplete those resources in the brain that underpin PM, future research may wish to explore which of these 70 plus toxins contained in tobacco smoke interact with excessive alcohol use to produce a detrimental impact upon everyday memory.

## Conclusion and Limitations

To the best of our knowledge, this is the first study to examine the synergistic impact of combined excessive alcohol use and cigarette smoking upon everyday PM. The findings revealed that individuals who consumed excessive amounts of alcohol and also smoked cigarettes demonstrated significantly greater deficits in time-based PM than the combined deficits from the single use of either excessive alcohol or cigarette smoking, suggesting a synergistic interactive effect rather than an additive effect of these two substances. It is our hope that the findings uncovered here will help to improve our understanding about the dangers of excessive drinking and smoking beyond the mainly health concerns highlighted in the literature by providing a greater understanding of the cognitive consequences of such polydrug use. Specifically, highlighting the dangers of combined heavy alcohol use and smoking in relation to everyday memory, in this case PM.

There are a number of limitations that should be considered when interpreting the findings form this study. One limitation of the study is the reliance on self-reported drug use, which can be problematic given that it relies upon the honesty and accuracy of the individual. Although the RDUQ (used in the current study to measure substance use) has been used in several studies to measure alcohol, smoking, and other substance use (9–11, 18–20, 31); its utility when compared with other substance use measures has not been tested, which should be considered when considering its use in future studies. Future research should overcome this by utilizing biological drug-screening techniques that provide objective and more accurate measures of alcohol and other drug use, for example the use of blood, urine and hair assays. The study asked anyone who used other substances (such as cannabis, ecstasy, etc.) or who had suffered from/were currently suffering from, a clinical disorder (such as amnesia, depression, or substance dependence), to refrain from taking part in the study as a method of screening participants. However, these were not assessed by biological assays (for measures of drug use) or clinical testing (for clinical disorders). This can be seen as a limitation of the present study, particularly given the fact that there is evidence that polydrug use is associated with greater health risks than single drug use (37), that polydrug use is more prevalent in psychiatric populations (38) and is associated with elevated levels of psychiatric conditions, such as aggression and suicide (39), when compared with single user groups. In addition, given that polydrug users differ from single drug user groups in terms of personality factors, such as exhibiting higher levels of impulsivity and a greater propensity for risk taking (40–42), personality factors should also be taken into account in future work. Therefore, future research should utilize biological methods to more accurately assess substance use, as well as include health, personality, and psychiatric indices to compile a fuller picture of how polydrug users and single drug users differ on these domains and to measure what impact, if any, these domains might have upon everyday memory in the form of PM.

Although the present study has uncovered a synergistic effect of excessive alcohol use and smoking upon PM, future research should attempt to replicate these findings using more ecologically valid PM tasks. This could be achieved by the use of real-world PM tasks such as remembering to carry out an activity after a period of time has passed (e.g., remember to text the researcher 24 h following the completion of the study) or the use of the diary method, both of which have proven useful in measuring PM deficits in clinical populations (43) or the recent use of virtual reality techniques to measure PM (44). Finally, given the frequency with which adolescents drink heavily and smoke, future research should investigate the impact of heavy drinking and smoking in the period of adolescence to upon the developing adolescent brain (45). Since tobacco smoking and alcohol use are two of the most widely used drugs in the Western world; and given the fact that they inflict a great deal of harm upon society and the fact that these two drugs are often used concurrently, a much greater understanding is needed with regards the cognitive consequences of combined cigarette smoking and excessive alcohol use.

## Author Contributions

A-MM: manuscript, research plan development, data gathering, and analysis. TH: manuscript, administration, and editing. CH: manuscript, editing, and analysis.

## Conflict of Interest Statement

The authors declare that the research was conducted in the absence of any commercial or financial relationships that could be construed as a potential conflict of interest.
